# Risk factors for liner wear and head migration in total hip arthroplasty: a systematic review

**DOI:** 10.1038/s41598-023-42809-4

**Published:** 2023-09-20

**Authors:** Filippo Migliorini, Nicola Maffulli, Marco Pilone, Andreas Bell, Frank Hildebrand, Christian Konrads

**Affiliations:** 1 Department of Orthopaedic, Trauma, and Reconstructive Surgery, RWTH University Hospital, Pauwelsstraße 30, 52074 Aachen, Germany; 2 Department of Orthopaedic and Trauma Surgery, Academic Hospital of Bolzano, Teaching Hospital of Paracelsus Medical University (PMU), 39100 Bolzano, Italy; 3 Department of Orthopaedic and Trauma Surgery, Eifelklinik St.Brigida, Simmerath, Germany; 4 Department of Orthopaedic and Trauma Surgery, Hospital Sant’Andrea, University of Rome La Sapienza, Rome, Italy; 5 School of Pharmacy and Bioengineering, Keele University Faculty of Medicine, Stoke on Trent, ST4 7QB England; 6 Queen Mary University of London, Barts and the London School of Medicine and Dentistry, Centre for Sports and Exercise Medicine, Mile End Hospital, London, E1 4DG England; 7 Residency Program in Orthopaedics and Traumatology, University of Milan, 20122 Milan, Italy; 8 Department of Orthopaedics and Traumatology, Helios Hanseatic Hospital Stralsund, 18435 Stralsund, Germany; 9 Medical Faculty, University of Tübingen, 72076 Tübingen, Germany

**Keywords:** Medical research, Experimental models of disease

## Abstract

Total hip arthroplasty (THA) is a successful orthopaedic surgical procedure, and its longevity depends on bearing components and implant fixation. Optimizing polyethylene and ceramics has led to improved wear parameters and contributed to improved long-term outcomes. The present systematic review investigated whether time span from implantation, patient characteristics and performance status exert an influence on liner wear and head migration in THA. This study was conducted in conformity to the 2020 PRISMA guidelines. All the clinical investigations which reported quantitative data on the amount of liner wear and head migration in THA were considered. Only studies which reported quantitative data at least on one of the following patient characteristics were suitable: mean age, mean BMI (kg/m^2^), sex, side, time span between the index THA and the last follow-up (months) were eligible. A multiple linear model regression analysis was employed to verify the association between patient characteristics and the amount of liner wear and/or head migration. The Pearson Product-Moment Correlation Coefficient was used to assess the association between variables. Data from 12,629 patients were considered. The mean length of the follow-up was 90.5 ± 50.9 months. The mean age of patients at surgery was 58.4 ± 9.4 years, and the mean BMI was 27.2 ± 2.5 kg/m^2^. 57% (7199 of 12,629 patients) were women, and in 44% (5557 of 12,629 patients) THAs were performed on the left. The mean pre-operative Harris hip score was 46.5 ± 6.0 points. There was evidence of a moderate positive association between the amount of liner wear and the time elapsed between the index surgery to the follow-up (P = 0.02). There was evidence of a moderate positive association between the amount of head migration and the time elapsed between the index surgery to the follow-up (P = 0.01). No further statistically significant association was found. The time elapsed between the index surgery to the follow-up was the most important factor which influence the head migration and liner wear in THA. Patients’ characteristics and preoperative physical activity did not influence the amount of head migration and liner wear.

## Introduction

Total hip arthroplasty (THA) is a successful orthopaedic surgical procedure^1–4^. The longevity of an implanted hip prosthesis depends on bearing size and materials^5,6^. Wear consumption is the most frequent cause of THA failure^7–9^. The pattern of wear loss is classically described as biphasic^10^. The first phase last up to 24 months, and is named bedding-in Ref.^10^. In this phase, penetration of the femoral head in the acetabular component is progressive^11^. The second phase is the steady state, in which the consumption of wear is relatively slow^11^. The optimisation of polyethylene, metals and ceramics used as bearing materials for hip arthroplasty has led to improved wear parameters and contributed to improved long-term outcomes^12–16^. Different combinations of wear materials determine the different mechanical characteristics of the arthroplasty^17–19^. The harder the material, the lower the surface roughness and the lesser the vulnerability to deformation forces^20–22^. The diameter of the femoral head plays a crucial role in wear: small femoral heads present less wear consumption because friction is reduced while large femoral heads have greater stability and lower dislocation rate^23,24^. Positioning of the acetabular component is another factor that influences the wear rates^25,26^. The acetabular component should be positioned within 40° to 50° of abduction and between 10° to 15° of anteversion^17,27–31^. Chemical reactions induce degradation of the bearing component^32^. Oxidation of polyethylene, which can be induced by sterilisation, reduces the strength, ductility, and resistance of this material^33^. The use of vitamin E as an antioxidant reduces this problem^34^. Zirconium, present in ceramic components, undergoes in vivo transformation in two other crystalline phases^35–38^. This phenomenon increases surface roughness and, consequently, the wear rate^32^. Although the choice of implant is often based on avoiding short-term complications, such as dislocation, surgeons must consider long-term complications, such as aseptic loosening and periprosthetic fracture that can be influenced by material wear^23^. In the modern times of pre-rehabilitation and patient education, more detailed information about individual patient factors influencing the long-term survival of THA is needed. The implant that best fits the patients is the goal to aim. The analysis of how the demographic characteristic of the patient influence the final outcome is fundamental for better results. The present systematic review investigated whether time span from implantation, patient characteristics, and preoperative performance status exert an influence on liner wear and head migration in THA.

## Methods

### Eligibility criteria

All the clinical investigations which reported quantitative data on the amount of liner wear and head migration in THA were considered. Only studies which reported quantitative data on at least one of the following patient characteristics were deemed suitable: mean age, mean BMI (kg/m^2^), sex, side, time span between the index THA and the last follow-up (months) were eligible. Missing quantitative data under the outcomes of interests warranted exclusion of the study. The grey literature was not accessed. According to the author’ language capabilities, articles in English, German, Italian, French and Spanish were eligible. Only studies with level I to III of evidence, according to Oxford Centre of Evidence-Based Medicine^39^, were considered. Although opinions, letters, reviews, and editorials were not eligible, their qualitative findings were collected and reported in the discussion of the present study. Animals, in vitro, biomechanics, computational, and cadaveric studies were not eligible. Studies on revision setting, or studies which evaluated multiple joint arthroplasties, were not included, nor were those who enhanced the surgery with cell therapies (e.g. platelet rich plasma, mesenchymal stem cells). Studies which evaluated experimental implant design or rehabilitation protocols were also not eligible.

### Search strategy

This study was conducted according to the Preferred Reporting Items for Systematic Reviews and Meta-Analyses: the 2020 PRISMA statement^40^. The PICOT algorithm was preliminary pointed out:

- P (Problem): end-stage OA;
- I (Intervention): THA;
- C (Comparison): time span from THA, patient characteristics and performance;
- O (Outcomes): liner wear, liner wear/year, head migration.

In July 2023, the following databases were accessed: PubMed, Web of Science, Google Scholar, Embase. No time constrain was set for the search. The following matrix of keywords were used in each database to accomplish the search using the Boolean operator AND/OR: (THA OR total hip) AND (arthroplasty OR replacement OR prosthesis) AND (wear OR migration OR creep OR liner OR head). No additional filters were used in the databases search.

### Selection and data collection

Two authors (F. M. and A. B.) independently performed the database search. All the resulting titles were screened by hand and, if suitable, the abstract was accessed. The full-text of the abstracts which matched the topic were accessed. If the full-text was not accessible or available, the article was not considered for inclusion. A cross reference of the bibliography of the full-text articles was also performed by hand. Disagreements were debated and mutually solved by the authors. In case of further disagreements, a third senior author (N.M.) took the final decision.

### Data items

Two authors (F.M. and A.B.) independently performed data extraction. The following generalities were extracted: author, year of publication, length of the follow-up, and number of procedures. The following data concerning patient demographic were extracted: mean age, mean BMI, percentage of women, percentage of left side, mean preoperative Harris Hip Score (HHS)^41^. Data on the following outcomes of interest were extracted: mean liner wear (mm), mean liner wear per year (mm/year), mean head migration (mm).

### Assessment of the risk of bias and quality of the recommendations

The risk of bias were evaluated in accordance with the guidelines in the Cochrane Handbook for Systematic Reviews of Interventions^42^. Two reviewers (F.M. and A.B.) evaluated the risk of bias of the extracted studies independently. Disagreements were solved by a third senior author (N.M.). All the included studies were evaluated using the risk of bias of the software Review Manager 5.3 (The Nordic Cochrane Collaboration, Copenhagen). The following endpoints were evaluated: selection, detection, performance, attrition, reporting, and other bias.

### Synthesis methods

The statistical analyses were performed by the main author (F.M.) following the recommendations of the Cochrane Handbook for Systematic Reviews of Interventions^42^. For descriptive statistics, mean and standard deviation were used. To evaluate baseline comparability of patient demographic, the SPSS software was used. For the statistical analyses, the STATA/MP software (Stata Corporation, College Station, Texas, USA) was used. A multiple linear model regression analysis was performed to investigate whether an association between patient characteristics and the amount of liner wear and/or head migration exist. The Pearson Product-Moment Correlation Coefficient (r) was used. The Cauchy–Schwarz formula was used for inequality: + 1 is considered as positive linear correlation, while and − 1 a negative one. Values of 0.1 <|r|< 0.3, 0.3 <|r|< 0.5, and |r|> 0.5 were considered to have weak, moderate, and strong correlation, respectively. The overall significance was assessed through the χ^2^ test, with values of P < 0.05 considered statistically significant.

## Results

### Study selection

**Figure 1:**
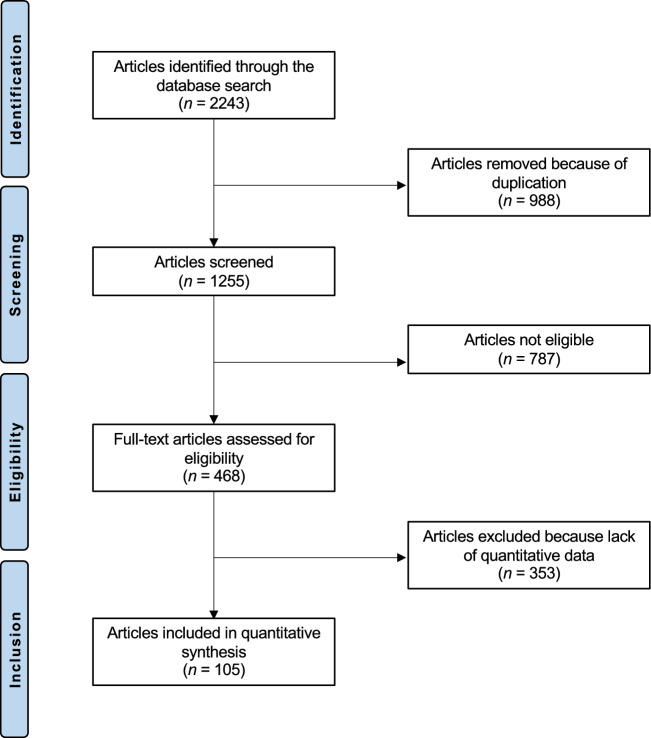
PRISMA flow chart of the literature search.

The initial databases search resulted in 2038 studies. Of them, 988 were duplicates. A further 787 studies were excluded with reason: study design (N = 326), not clinical investigations (N = 201), poor level of evidence (N = 184), not reporting any data of interest on patient characteristics (N = 39), revision setting, multiple joint arthroplasties, enhanced the surgery with cell therapies (N = 22), evaluating experimental implant design or rehabilitation protocols (N = 9). Language limitations (N = 6), A further 353 studies were excluded as they did not report quantitative data under the outcome of interest. Finally, 105 studies were included: 25 randomised controlled trials, 47 prospective and 33 retrospective clinical investigations. The results of the literature search are shown in Fig. 1.

### Risk of bias assessment

**Figure 2:**
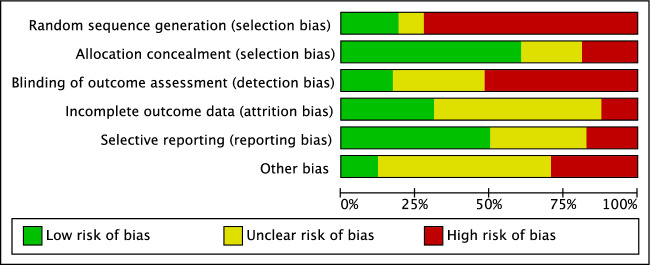
Cochrane risk of bias tool.

The Cochrane risk of bias tool was used to investigate between studies risk of bias. 24% (25 of 105) of included studies randomly allocated their patients, and 69% (72 of 105 studies) were conducted in a prospective fashion leading to a low to moderate risk of selection bias. The risk of detection bias was high, as assessor blinding was seldom performed. The risk of attrition and reporting biases was low to moderate, as was the risk of other bias. Concluding, the risk of bias graph evidenced a moderate quality of the methodological assessment of RCTs (Fig. 2).

### Study characteristics and results of individual studies

Data from 12,629 patients were considered in the present study. The mean length of the follow-up was 90.5 ± 50.9 months. The mean age of patients was 58.4 ± 9.4 years, and the mean BMI was 27.2 ± 2.5 kg/m^2^. 57% (7199 of 12,629 patients) were women, and 44% (5557 of 12,629 patients) were performed on the left side. The mean pre-operative HHS was 46.5 ± 6.0 points. The generalities and patient demographic of the included studies is shown in detail in Table 1.

**Table 1:** Generalities and patient baseline of the included studies (*RCT* randomised controlled trial).

Author	Year	Design	Follow-up (months)	Procedures (n)	Mean age	Mean BMI	Women (%)	Left side (%)	Mean HHS
Afghanyar et al.^43^	2021	Prospective	79	101	69.4	27.5			50
Busch et al.^44^	2020	RCT	60	43	62.3	28.5	56		
			60	51	62.3	28.5	54		
Heijnens et al.^45^	2020	Prospective	172	29	55.4	27.2	48	45	
Kim et al.^46^	2020	Prospective	205	133	53.0	28.0	37		39
			205	133	53.0	28.0	37		41
Kjærgaard et al.^47^	2020	RCT		24	65.0	28.0	21		47
				29	63.0	29.0	31		49
				30	64.0	28.0	36		50
				33	61.0	27.0	42		44
Massier et al.^48^	2020	Prospective	72	102	66.0		75		
			72	97	65.0		66		
Moon et al.^49^	2020	Retrospective	208	22	49.7	23.5	45	39	
			185	112	52.3	23.5	50	46	
Pallante et al.^50^	2020	Retrospective	96	53	17.0	26.0	47	48	
			96	28	17.0	26.0	47	48	
			96	10	17.0	26.0	47	48	
Rochcongar et al.^51^	2020	RCT		33	61.0	27.0	48		52
				29	61.0	27.0	52		53
Thoen et al.^52^	2020	RCT		37	58.0	28.5	46	54	49
				31	61.0	26.6	48	61	51
van Loon et al.^53^	2020	RCT	60	25	68.5	26.7	39		51
			60	26	68.6	27.2	59		55
Bryan et al.^54^	2019	Retrospective		216	42.6	29.6			46
				57	40.1	26.3			46
Feng et al.^55^	2019	Prospective	86	77	59.0	23.2	43		40
			83	93	51.0	25.2	43		48
Galea et al.^56^	2019	Prospective		39	66.1	27.2	56		
				34	62.6	28.3	59		
Sköldenberg et al.^57^	2019	Prospective		21	67.0	27.0	48	33	48
				21	67.0	27.0	52	29	41
Atrey et al.^58^	2018	Prospective	180	28	41.5	26.7	50		50
			180	29	42.8	28.2	55		49
Galea et al.^59^	2018	Prospective	60						
			60						
			60						
			60						
Gaudiani et al.^60^	2018	Retrospective	72		59.0	28.1	62	43	
			78		52.9	27.0	62	38	
Higuchi, et al.^61^	2018	Retrospective	79	77	64.7	23.1	88		57
			80	105	55.9	23.0	81		60
Hopper et al.^62^	2018	Prospective	188	116	62.5	28.6	56		
			176	114	62.0	27.9	50		
Mayer et al.^63^	2018	Prospective	109	72	46.5	26.4	56		
Morrison et al.^64^	2018	Prospective	139	20	81.7	26.2	70		
			140	18	80.6	32.6	72		
Teeter et al.^65^	2018	Retrospective	61	20	57.1	30.4	80		
			67	20	57.2	31.0	80		
			62	18	59.9	31.0	44		
			65	18	60.1	35.2	44		
Atrey et al.^66^	2017	RCT	120	29					49
			120	34					49
			120	29					46
Broomfield et al.^67^	2017	Prospective	146	27	68.0		45		
			146	27	67.0		53		
Kawata et al.^68^	2017	Prospective		26	60.0				
				25	61.5				
				23	62.6				
				20	60.8				
Nebergall et al.^69^	2017	Prospective		32	67.0	27.0	50		59
				35	65.0	27.0	54		52
Rajpura et al.^70^	2017	Prospective	330	9	46.6				
Scemama et al.^71^	2017	Prospective		50	66.0	26.0	48		
				50	67.0	25.0	56		
Takada et al.^72^	2017	Retrospective	64	54	60.1	22.5	89		
			64	55	65.5	23.2	84		
Teeter et al.^73^	2017	RCT	156	8	67.5	28.4			38
			156	8	67.5	28.4			37
Tsukamoto et al.^74^	2017	Retrospective	150	41	56.3		93		36
			156	38	57.9		89		34
Hamai et al.^75^	2016	Retrospective	121	36	61.1		86		
			121	36	60.7		86		
Hanna et al.^76^	2016	Retrospective	158	89	56.8	30.7	51		
			157	88	55.6	30.0	90		
Higuchi et al.^77^	2016	Retrospective	132	67	54.0	23.9	78		56
			136	81	54.2	22.5	83		55
Sato et al.^78^	2016	Retrospective	228	110	60.3	20.4	85		
			241	73	59.8	22.0	85		
Sillesen et al.^79^	2016	Retrospective		520	60.8	28.4	50		51
				457	62.3	28.5	50		50
Ayers et al.^80^	2015	Prospective	60	11	58.0	29.0	73		41
			60	12	56.0	30.0	67		46
			60	11	59.0	28.0	45		46
			60	12	60.0	31.0	50		46
Garvin et al.^81^	2015	Prospective	108	19	42.0	30.0			
			108	34	42.0	30.0			
			108	43	42.0	30.0			
Glyn-Jones et al.^82^	2015	Prospective	120	19	67.0		53		
			120	20	68.0		45		
Jassim et al.^83^	2015	Prospective	60	123	61.0		66		
			60	121	63.0		56		
			60	124	63.0		56		
Jonsson et al.^84^	2015	Prospective		30	69.0	27.0	67	50	41
				30	69.0	26.0	77	60	47
				30	70.0	27.0	67	47	47
				30	70.0	27.0	73	40	40
Keeney et al.^85^	2015	Retrospective		84	40.4	28.8	43		38
				89	40.3	27.7	58		45
Langlois et al.^86^	2015	Prospective		50	66.4	24.4	55		
				50	66.4	24.4	55		
Pang et al.^87^	2015	Retrospective		13	61.0	32.0	62	38	
				13	66.0	32.0	62	38	
Shareghi et al.^88^	2015	Prospective		38	58.0	25.0	42		43
				32	58.0	27.0	53		46
Epinette et al.^89^	2014	Retrospective	126	228	68.7	28.1	66		44
			135	447	68.0	27.4	68		40
Morison et al.^90^	2014	RCT	82	21	50.6	30.3	48		45
			82	23	53.7	27.9	48		46
			82	21	52.4	27.1	36		43
			82	22	51.2	29.3	55		49
Topolovec et al.^91^	2014	Retrospective		26	68.0		92		
				12	74.0		67		
Dahl et al.^92^	2013	Retrospective	120	23	60.0		74	48	52
			120	20	64.0		55	40	55
Fukui et al.^93^	2013	Retrospective	125	36	56.7	23.1	94		
			127	20	53.0	22.7	80		
García-Rey et al.^94^	2013	Prospective		42	67.4		57		
				41	61.1		54		
Hasegawa et al.^95^	2013	Prospective	84	23	64.0	24.1	91		
			84	68	57.0	23.2	91		
Kim et al.^96^	2013	Prospective	149	100	45.3		50		38
			149	100	45.3		50		37
Nakashima et al.^97^	2013	Retrospective	157	62	62.0	23.9	70		
			138	69	61.8	24.3	82		
Vendittoli et al.^98^	2013	RCT	148	69	56.8	27.3	45	45	
			148	71	54.9	28.2	58	46	
Wang et al.^99^	2013	Retrospective	120	22	51.5		50		
			120	22	51.5		50		
Engh et al.^100^	2012	RCT		116	62.5	28.6	56		
				114	62.0	27.9	50		
Johanson et al.^101^	2012	Prospective		27	56.0		44		46
				25	55.0		52		44
Nikolaou et al.^102^	2012	RCT	60	36	52.6	28.7	50		47
			60	32	55.1	32.6	56		52
			60	34	52.0	28.2	50		46
Sato et al.^103^	2012	Retrospective	145	40	59.6		63		
			145	24	59.6		56		
			73	275	61.8		85		
			73	72	61.8		85		
			73	20	61.8		85		
Amanatullah et al.^104^	2011	Prospective		196	50.4	29.6	36		
				161	54.7	28.0	43		
Mall et al.^105^	2011	Retrospective	72	50	43.2				
			99	48	46.5				
Orradre Burusco et al.^106^	2011	Prospective	65	50	65.4	25.5	36	44	36
			70	57	67.6	25.6	40	44	39
Thomas et al.^107^	2011	Prospective	84	22	68.0		55		
			84	22	67.0		50		
Huddleston et al.^108^	2010	Prospective	128	45	57.0	27.1	26		55
			120	43	60.0	25.4	43		57
Lewis et al.^109^	2010	RCT	120	23	42.8	28.2			
			120	23	41.5	26.7			
Mutimer et al.^110^	2010	RCT	66	55	61.0		53		
			66	55	62.0		36		
Nakahara et al.^111^	2010	Prospective	80	47	57.5	23.5	81		
			79	47	56.9	23.5	87		
Beksaç et al.^112^	2009	Retrospective	64	41	50.0	28.0	43		
			64	41	53.0	30.0	43		
Calvert et al.^113^	2009	RCT		60	62.5		45	42	49
				59	61.0		59	42	52
Geerdink et al.^114^	2009	RCT	96	26	64.0	28.0	43		40
			96	22	64.0	28.0	35		39
Hernigou et al.^115^	2009	Retrospective	240	28	55.0				
			240	28	55.0				
Ise et al.^116^	2009	RCT	48	26	60.0		96		
			46	25	61.6		94		
			45	23	62.7		100		
			49	20	60.9		94		
Kawate et al.^117^	2009	RCT							43
									47
Kim et al.^118^	2009	Prospective	67	100	45.3	23.0	34		39
			67	100	45.3	23.0	34		41
McCalden et al.^119^	2009	RCT	80	50	72.6	29.7	72		39
			84	50	72.3	29.7	66		36
Rajadhyaksha et al.^120^	2009	Retrospective	71	27	60.3	27.6	32		59
			75	27	62.0	28.1	44		49
Shia et al.^121^	2009	Retrospective	48	70	41.0		46	49	53
Stilling et al.^122^	2009	Retrospective	58	36	53.5		15	50	54
			58	33	51.5		42	42	57
			85	54	44.2		11		41
			85	54	44.2		11		43
Bitsch et al.^123^	2008	Retrospective	69	32	60.0	30.5	69		
			70	24	74.0	27.3	54		
García-Rey et al.^124^	2008	RCT	66	45	60.6				
			66	45	62.5				
Glyn-Jones et al.^125^	2008	RCT	24	26	68.0				
			24	26	67.0				
			24	26	68.0				
			24	26	67.0				
Miyanishi et al.^126^	2008	Retrospective	28	95	67.0	24.7	83		
			50	20	61.0	24.8	79		
Digas et al.^127^	2007	Prospective			55.0		100		
					55.0		100		
				32	48.0		66		
				32	48.0		66		
Ise et al.^128^	2007	Prospective	80	46	58.1		88		
			65	50	58.3		94		
Kim et al.^129^	2007	Prospective	58	50	51.0		24		
			58	50	51.0		24		
Röhrl et al.^130^	2007	Prospective	60	20	70.0		40	20	43
			72	10	58.0		40	33	47
Triclot et al.^131^	2007	RCT	60	33	67.9	26.5	48		
			60	34	70.1	26.4	41		
Vendittoli et al.^132^	2007	RCT	79	69	56.8		45	45	
			79	71	54.9		58	46	
Bragdon et al.^133^	2006	Prospective	45	41	60.3				
			45	12	60.3				
			45	70	60.3				
Engh et al.^134^	2006	Prospective	68	116	62.5	28.6	56		
			68	114	62.0	27.9	50		
Geerdink et al.^135^	2006	Prospective	56	54	63.0	27.0			
			56	45	64.0	28.0			
Kraay et al.^136^	2006	RCT	52	30	68.9		65		51
			51	27	69.5		74		48
Oonishi et al.^137^	2006	Prospective	28	70	61.0				
			28	73	61.0				
Zhou et al.^138^	2006	Prospective		31	66.0		68		
				30	68.0		47		
D'Antonio et al.^139^	2005	Retrospective	59	56	57.4	26.9	49		
			64	53	52.9	27.5	42		
Dorr et al.^140^	2005	Prospective	60	37	60.2		54		
			60	37	65.1		54		
Krushell et al.^141^	2005	Retrospective	48	40	68.7	27.9	53		
			50	40	69.5	28.2	53		
Manning et al.^142^	2005	Prospective		111	57.0	25.6	44		
			44	70	60.9	25.9	50		
Röhrl et al.^143^	2005	Prospective	24	20	70.0		40		43
			24	20	67.0		75		47
			36	10	58.0		40		43
Digas et al.^144^	2004	RCT		27	48.0		63		42
				27	48.0		63		44
				23	55.0		57		49
				26	57.0		46		47
Hopper et al.^145^	2003	Retrospective	37	78	58.7				
			36	50	60.3				
			35	48	60.3				
			34	50	61.0				
			28	24	60.0	30.6			
			28	22	55.0	27.6			
Pabinger et al.^146^	2003	RCT	24	31			39		
			24	28			43		
Kim et al.^147^	2001	Prospective		35	39.9		17		
				35	39.9		17		
				35	39.9		17		
				35	39.9		17		

### Synthesis of results

There was evidence of a moderate positive association between the amount of wear and the time elapsed between the index surgery and the last follow-up (*r* = 0.22; P = 0.02). There was evidence of a moderate positive association between the amount of migration and the time elapsed between the index surgery to the last follow-up (*r* = 0.57; P = 0.01). No statistically significant association was found between the amount of wear and patient age (P = 0.2), BMI (P = 0.4), sex (P = 0.3), side (P = 0.4), and pre-operative HHS (P = 0.05). No statistically significant association was found between the amount of migration and patient age (P = 0.6), BMI (P = 0.3), sex (P = 0.6), side (P = 0.3), and pre-operative HHS (P = 0.1). No statistically significant association was found between the amount of wear per year and patient age (P = 0.1), BMI (P = 0.5), sex (P = 0.1), side (P = 0.8), pre-operative HHS (P = 0.6), and the time elapsed between the index surgery to the follow-up (P = 0.3). These results are shown in greater detail in Table 2.

**Table 2:** Results of the linear regressions.

Item	Age		BMI		Female sex		Left side		Follow-up		HHS	
	*r*	P	*r*	P	*r*	P	*r*	P	*r*	P	*r*	P
Wear	− 0.1	0.2	0.1	0.4	− 0.1	0.3	0.2	0.4	0.2	0.03	0.1	0.05
Wear/year	− 0.1	0.1	0.1	0.5	− 0.1	0.1	− 0.2	0.8	0.1	0.3	0.1	0.6
Migration	− 0.0	0.6	− 0.1	0.3	0.0	0.6	0.1	0.3	0.6	0.01	− 0.1	0.1

## Discussion

According to the main findings of the present study, the time elapsed between the index surgery to the follow-up was the most important factor which influences head migration and liner wear in THA. Moreover, patient age, BMI, sex, side, and preoperative HHS did not exert an influence in the amount of head migration and liner wear. The postoperative activity level as a potential parameter affecting head migration and liner wear could not be analysed because of missing relevant data in this regard.

The use of conventional polyethylene versus highly cross-linked polyethylene (HXPE) or vitamin E-infused highly cross-linked polyethylene (VE-HXPE) leads to higher wear rates and shorter implant survival, whereas no difference could be found between HXPE and VE-HXPE materials^34^. A recent study on 137 patients showed less wear rate in HXPE THA than in conventional polyethylene THA (0.028 mm/year and 0.086 mm/year, respectively)^49^. Survival rate after 18 years follow up was 95.5% in the HXPE group and 90.9% in the conventional polyethylene group^49^. In a randomised controlled trial study on 94 patients, 51 received a VE-HXPE THA and 43 received a HXPE THA^44^. After 5 years, there was no statistically significant difference in wear rate (24.0 μm/year in VE-HXPE group and 23.2 μm/year in HXPE group). VE-HXPE demonstrated better results than HXPE after 10 years follow-up given the reduction of oxidative embrittlement. In general, HXPE, VE-HXPE, or ceramic on ceramic components exhibit the best wear and life span properties^47^. A positive association between the time elapsed between the index surgery to the follow-up and the amount of wear migration was evidenced. Migration results from the plastic deformation of polyethylene that occurs during the first 12–24 months, known as the bedding-in period^148,149^. The duration of the migration phase is debated. It is probably an overlapping process, time-dependent, as confirmed by our results^150^. Eliminating migration from total wear estimation resulted in an adjusted value that was nearly 50% lower than previously estimated total wear values^151^.

Metal on metal bearings lead to higher amounts of metal ions in the surrounding tissue and serum^152^. This could be seen as an indirect sign of component wear^153^, and leads to local inflammation, which promotes implant loosening through osteolysis^154^. Additionally, the metal ions can produce toxic systemic complications and deterioration of organ functions^155^. Metal on metal bearings is no longer recommended given these effects^156^.

Metal heads can be safely used with a polyethylene liner^157^. It is not clear whether any difference exists using metal head or ceramic head with HXPE^158,159^. Guadiani et al.^60^ in a study on 120 patients showed a wear rate of 0.0135 mm/year using a ceramic head and 0.0171 mm/year using metal head. No differences were found in functional scores.

The most common materials are ceramic on ceramic, ceramic on polyethylene and metal on polyethylene^32^. A randomised controlled trial analysed the long-term functional and radiographic outcomes in 133 patients after bilateral THA^46^. In one hip, a ceramic-on-ceramic THA was implanted and in the other hip a ceramic-on-highly cross-linked polyethylene was implanted. After 17.1 years of follow-up, the functional results were comparable with no signs of osteolysis observed in either group.

Van Loon et al.^160^ conducted a 10 years follow-up study analysing factors that can predict wear in ceramic-on-ceramic and ceramic-on-polyethylene THA. In accordance with our results, they showed that BMI, age and gender do not influence wear rate. Another study with 20 years follow-up confirms these results^161^. Garvin et al.^81^ showed a very low wear rate in patients under 50 years old, at 0.022 mm/year. A recent study conducted by Sax et al.^162^ on 130 THAs, using second-generation highly cross-linked polyethylene THA, showed opposite results, identifying an association between age and volumetric wear and an association between BMI and volumetric and linear wear. Younger patients have higher activities level than older patients^163^, but 10 years follow up study demonstrated that sport activities have no influence on migration and wear rate^164^. Low impact sport activities such as walking were included in the study^164^. There is an increasing number of young patients who undergo a THA, and the positive effect of sport on health and quality of life is well demonstrated^165,166^. Guy et al.^167^ analysed wear rate in patients who practised high-impact sports. 34 patients received a ceramic on HXLPE implant, and 34 patients received ceramic on conventional polyethylene implant. The HXLPE group showed a statistically significant lower wear rate and osteolysis rate than the conventional polyethylene group. Consensus guidelines for returning to sport after THA suggested that return to sport should be allowed for low-impact and moderate-impact sports, but not for high-impact sports^168^. The patients' main reason not to return to sport was surgeon's advice^169^. However, no difference in revision rate was found when comparing a sporting population with less active controls^170,171^. Two studies comparing obese with non-obese patients did not show an association between BMI and aseptic loosening, although the higher the BMI, the higher the reactive force through the hips^172,173^.

A major strength of the present study is the comprehensive analysis of the main demographic factors that can influence liner wear and head migration. To our knowledge, no other study examined the effect of these variables on THA, including all types of materials. The presence of a large number of RCTs in our study strengthens our results. Given the lack of quantitative data, it was not possible to analyse all the possible combinations of head and liner materials. The grey literature, i.e. unpublished or non-peer-reviewed research, was not included in the present study. It would be difficult to locate and assess for quality. Heijnens et al.^45^ presented disappointing long-term results because of aseptic loosening in four of their 29 patients using carbon-fibre-reinforced poly-ether-ether-ketone (CFR-PEEK) liners, which might have influenced our results. Some studies did not differentiate between patients who had unilateral or bilateral THA. In unilateral THA, the forces distributed unequally between the two joints. Moreover, frequently the contralateral side is osteoarthritic and symptomatic. A painful contralateral hip, knee, or ankle might lead to increased weight-bearing of the operated leg. This could not be appreciated in most studies analysed for this systematic review. The size of the femoral head is another factor that can influence wear rate and migration: unfortunately, this could not be analysed given the lack of relevant data. Further investigations are necessary to investigate the association between liner wear and sport load.

## Data Availability

The datasets generated during and/or analysed during the current study are available throughout the manuscript.
